# Lack of Hypothalamus Polysialylation Inducibility Correlates With Maladaptive Eating Behaviors and Predisposition to Obesity

**DOI:** 10.3389/fnut.2018.00125

**Published:** 2018-12-10

**Authors:** Xavier Brenachot, Emmanuelle Nédélec, Selma Ben Fradj, Gaelle Boudry, Véronique Douard, Amélie Laderrière, Aleth Lemoine, Fabienne Liénard, Danaé Nuzzaci, Luc Pénicaud, Caroline Rigault, Alexandre Benani

**Affiliations:** ^1^Centre des Sciences du Goût et de l'Alimentation, AgroSup Dijon, CNRS, INRA, Université de Bourgogne, Dijon, France; ^2^Institut NuMeCan, INRA, INSERM, Université Rennes, Domaine de la Prise, Saint-Gilles, France; ^3^Institut Micalis, INRA, AgroParisTech, Université Paris-Saclay, Domaine de Vilvert, Jouy-en-Josas, France

**Keywords:** food intake, obesity, maladaptive eating behavior, synaptic plasticity, PSA-NCAM, polysialylation, brain, hypothalamus

## Abstract

High variability exists in individual susceptibility to develop overweight in an obesogenic environment and the biological underpinnings of this heterogeneity are poorly understood. In this brief report, we show in mice that the vulnerability to diet-induced obesity is associated with low level of polysialic acid-neural cell adhesion molecule (PSA-NCAM), a factor of neural plasticity, in the hypothalamus. As we previously shown that reduction of hypothalamic PSA-NCAM is sufficient to alter energy homeostasis and promote fat storage under hypercaloric pressure, inter-individual variability in hypothalamic PSA-NCAM might account for the vulnerability to diet-induced obesity. These data support the concept that reduced plasticity in brain circuits that control appetite, metabolism and body weight confers risk for eating disorders and obesity.

## Introduction

Changes in lifestyle and in the availability and quality of food largely explain the worldwide epidemic of obesity (1). However, high variability exists in individual susceptibility to develop overweight in an obesogenic environment (2). This implies that genetic risk factors among individuals substantially influence the variability in body mass index (3). A better understanding of the molecular basis of this heterogeneity might help to fight against obesity and its related disorders. In the past, numerous animal models have been used to investigate pre-existing differences that are established before the onset of corpulence and that confer a risk for common obesity (4–7). In this way, prospective studies in rats have shown that some early metabolic responses to high fat diet (HFD) at normal weight are predictive of propensity to develop obesity and liver diseases on the long term (8–11). In the present study, we found in mice that aberrant feeding behavior in response to dietary fat constitutes a latent vulnerability trait to obesity. This predictive model can be relevant to identify biological risk factors for maladpative eating behaviors and obesity before the onset of the disease. Recent genetic studies in human indicated that most of the genes associated to the body mass index are enriched in the brain and related to neuronal biology and synaptic plasticity (12, 13). Thus, we further examined the expression of polysialic acid-neural cell adhesion molecule (PSA-NCAM), a marker of neural plasticity, in mice prone to obesity, and we found that the vulnerability to obesity was correlated with decreased hypothalamus level of PSA-NCAM.

## Methods

### Animals

Protocols including manipulation of animals (registration number 853.01) were reviewed and approved by our local Institutional Animal Care and Use Committee (“Comité d'éthique en expérimentation animale n°105”), and were in strict accordance with the European Community guidelines (directive 86/906). Experiments were carried out with 2 months-old male C57Bl/6JOla mice from Harlan Laboratories. Mice were housed individually, fed either a standard diet (catalog number A04; Safe Laboratories; Augy, France; 3.3 kcal/kg; energy from carbohydrate/fat/protein: 72.4/8.4/19.3) or a customized highly palatable high-fat diet (catalog number U8954P V.7; Safe Laboratories; 4.4 kcal/kg; energy from carbohydrate/fat/protein: 40.8/42.9/16.3). Free access was given for food and water. Recordings of food intake and body weight were done manually at 9:00 h. For tissue collection, mice were killed between 14:00 and 16:00 h after a 6-h fast.

### Medio-Basal Hypothalamus Dissection

After sacrifice, brains were quickly removed and immersed in cold PBS solution. A 2 mm coronal slice was cut with a brain matrix. The slice was laid on a 6% agarose block and the MBH was dissected under stereomicroscope and cold-light illumination using a scalpel. Tissues were immediately frozen in liquid nitrogen and stored at −80°C until experiments.

### PSA-NCAM Assay

Tissues were lysed and homogenized in RIPA lysis buffer using the TissueLyser system (Qiagen; Courtaboeuf, France) and 5 mm stainless steel beads (Qiagen). The homogenates were centrifuged 5 min at 5,000 *g* and supernatants were collected for PSA-NCAM assay using an enzyme-linked immunosorbent assay kit (PSA NCAM ELISA kit; Eurobio; Courtaboeuf, France) and for protein assay (Bio-Rad Protein Assay Kit ll; Biorad; Marnes-la-Coquette, France).

### Statistical Analysis

All data are expressed as means. Error bars indicate standard errors of the mean (SEM). Multiple comparisons between groups were carried out by one- or two-way ANOVA using Prism 5.0 software (GraphPad Software; San Diego, CA, United States). *Post-hoc* analyses were done when main effects reached significance. Before comparison, Bartlett's and Shapiro–Wilk's tests were applied to check equality of variances and to evaluate the normality of the distribution, respectively.

## Results

### The Homeostatic Feeding Response to a Dietary Fat Challenge Reveals Obesity Susceptibility

**Figure 1:**
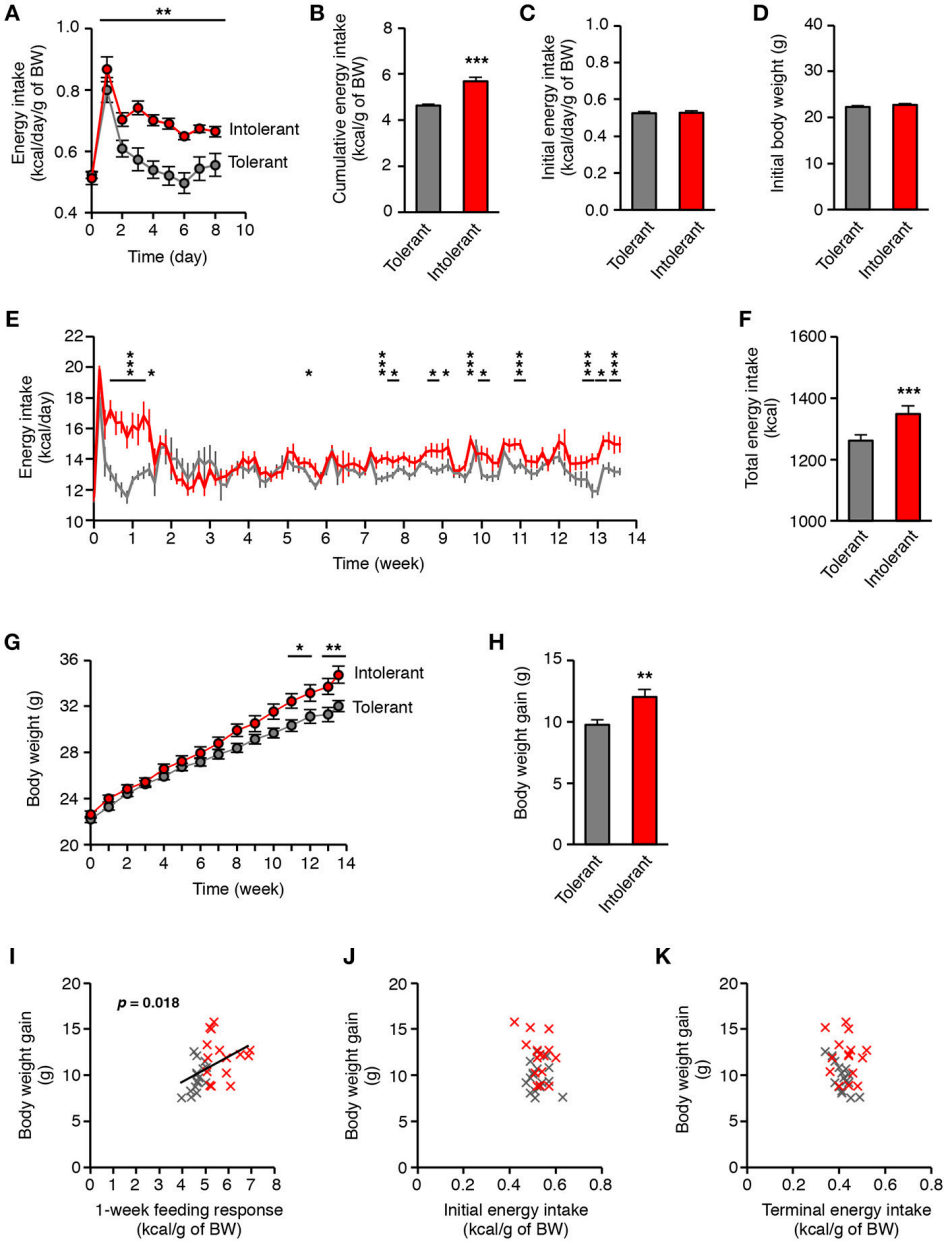
Individual differences in the homeostatic feeding response to HFD reveal susceptibility to diet-induced obesity. **(A)** Energy intake per gram of body weight of HFD-tolerant (*n* = 16) and HFD-intolerant (*n* = 15) mice during 1-week HFD (^**^p < 0.01, HFD-tolerant vs. HFD-intolerant mice; two-way ANOVA and Bonferroni *post-hoc* test). **(B)** Cumulative energy intake per gram of body weight of HFD-tolerant and HFD-intolerant mice after 1 week on HFD (^***^p < 0.001; Mann Whitney test). **(C,D)** Initial energy intake per gram of body weight and body weight of HFD-tolerant and HFD-intolerant mice were similar on standard chow, i.e., before HFD introduction. **(E)** Energy intake of HFD-tolerant and HFD-intolerant mice during 13 weeks-HFD (^*^p < 0.05, ^***^p < 0.001, HFD-tolerant vs. HFD-intolerant mice; two-way ANOVA and Bonferroni *post-hoc* test). **(F)** Total energy intake of HFD-tolerant and HFD-intolerant mice after 3-month HFD (^***^p < 0.001; unpaired *t*-test). **(G)** Body weight of HFD-tolerant and HFD-intolerant mice during 13-week HFD (^*^p < 0.05, ^**^p < 0.01, HFD-tolerant vs. HFD-intolerant mice; two-way ANOVA and Bonferroni *post-hoc* test). **(H)** Body weight gain of HFD-tolerant and HFD-intolerant mice after 13-week HFD (^**^p < 0.01; unpaired *t*-test). **(I)** Correlation analysis between the acute feeding response to HFD and the body weight gain after 3-month HFD. HFD-tolerant mice are in gray, HFD-intolerant mice are in red (*p* < 0.018; linear regression). **(J)** Correlation analysis between the initial energy intake on standard diet and the body weight gain after 3-month HFD. **(K)** Correlation analysis between the terminal energy intake on HFD and the body weight gain after 3-month HFD. All results are mean ± SEM.

For a 2-month old mice fed with standard diet, the typical daily energy intake is 0.5 kcal per gram of body weight (The Jackson Laboratory, Mouse Phenome Database, http://phenome.jax.org/). It is well-known that high-energy foods cause transient overeating in most of the mice, which corresponds to an acute increase in energy intake during a few days (14–16), as illustrated in Figure S1A. In this study, the period of overconsumption was variable between mice and normalization of energy intake occurred after 2 days or more (Figure S1B; in kcal/gram of body weight). Consequently, the individual cumulative energy intake during 1-week HFD, hereinafter referred to as the “feeding response,” ranged from 3.96 to 6.92 kcal/g (Figure S1C). According to this response, we identified by a median split HFD-intolerant mice with high feeding response due to slow normalization, and HFD-tolerant mice with low feeding response due to fast normalization (Figures 1A,B). The two groups of mice were initially undistinguishable with respect to their energy intake on standard diet or to their initial body weight (Figures 1C,D). On HFD, tolerant mice normalized their energy intake in only 2 days after HFD introduction, whereas intolerant mice normalized it after 10 days (Figure 1E; in kcal/gram of body weight). After 2 weeks on HFD, energy intake was normalized for all mice. Nevertheless, intolerant mice kept on HFD for a long term had further episodic increases in raw energy intake which appeared after 5 weeks on HFD (Figure 1E; in kcal). Therefore, the cumulative energy intake over 3 months with HFD was significantly higher in intolerant mice than in tolerant mice (Figure 1F). Moreover, the longitudinal follow-up showed that body weight of intolerant mice maintained on HFD increased strongly after 8 weeks in comparison to tolerant mice (Figure 1G). Hence, the body weight gain between the two groups of mice was significantly different after 3 months on HFD (Figure 1H). Importantly, the terminal body weight gain was positively correlated with the feeding response to HFD (Figure 1I). By contrast, it was not correlated with the initial energy intake on standard diet or with the terminal energy intake on HFD (Figures 1J,K). These results indicated that the homeostatic feeding response to an acute HFD challenge in mice is a latent trait predictive of the propensity to gain weight under persistent caloric pressure.

### Relation Between Fat Tolerance and Hypothalamus Polysialylation

Identification of individuals prone to obesity before the onset of the disease allows investigation of the biological underpinnings of susceptibility to obesity and makes possible the discovery of biological risk factors. Pre-existing differences in brain circuits controlling appetite and energy homeostasis might account for the vulnerability to common obesity (17, 18). Recent genetic studies formally implicated the brain in obesity pathology and pinpointed neuronal genes regulating synaptic plasticity (12, 19, 20). Moreover, rodent data consistently evidenced alterations of neural function and plasticity in brain feeding circuits in animals genetically predisposed to obesity (21, 22). Thus, we hypothesized that innate alteration in molecules involved in brain neuroplasticity would confer risk for obesity.

**Figure 2:**
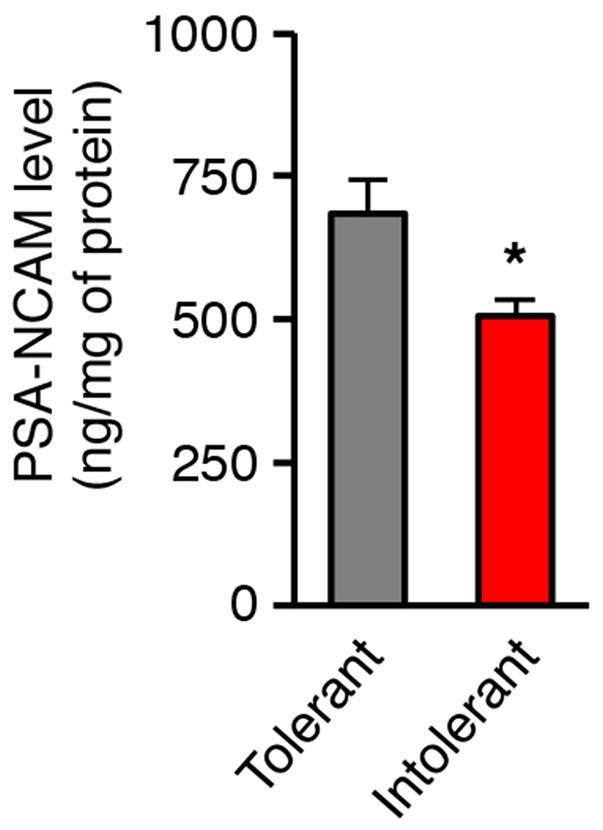
Individual differences in the tolerance to HFD are linked to the level of hypothalamic PSA-NCAM. PSA-NCAM levels were measured in mediobasal hypothalamus after a 1-week HFD challenge allowing identification of HFD-tolerant and HFD-intolerant mice (*n* = 7 HFD-tolerant, *n* = 7 HFD-intolerant; ^*^p < 0.05; Mann Whitney test). Results are mean ± SEM.

The polysialic acid-neural cell adhesion molecule (PSA-NCAM) is a cell-surface molecule that promotes various plasticity-related changes in brain circuits (23). We recently identified PSA-NCAM in the hypothalamus as a permissive factor for the brain control of energy balance (16, 24, 25). We therefore postulated that low level of hypothalamic PSA-NCAM would incite vulnerability to obesity. To test this hypothesis, we identified in a second cohort of mice (*n* = 14), HFD-tolerant and HFD-intolerant individuals according to their feeding response to 1-week HFD and then we examined the concentration of PSA-NCAM in the medio-basal hypothalamus (MBH) collected after this oral fat tolerance test. Intolerant mice showed significant lower levels of hypothalamic PSA-NCAM in comparison to tolerant mice (Figure 2; tolerant: 684.7 ± 59.4 ng/mg; intolerant: 505.9 ± 27.1 ng/mg; *p* = 0.038). As a reference, the constitutive level of PSA-NCAM in the MBH of control mice fed a standard diet was 508.5 ± 31 ng/mg protein (*n* = 16). The level of PSA-NCAM in the MBH of intolerant mice did not differ from the constitutive value. These data suggest that tolerant mice might have higher ability to mobilize PSA-NCAM to trigger synaptic plasticity in the hypothalamus than intolerant mice.

## Discussion

In this study, we measured the feeding response to 1-week HFD in mice. We found variability in the behavioral response with high responders who failed to rapidly normalize their energy intake during the metabolic challenge. High responders also gained more weight when they consumed the hypercaloric diet for a long time. Thus, the 1-week HFD challenge may be considered as a Dietary Fat Tolerance Test (DFTT) for identifying mice prone to nutritional obesity. Similar predictive tests have been used in the past showing that short-term response to HFD constitutes a phenotypic trait of metabolic flexibility which provides information on vulnerability to metabolic diseases (8–11). The present study further shows that the DFTT could be based on the behavioral response only, and does not necessarily imply collecting biopsies for metabolic investigation.

The DFTT might be relevant for investigating intrinsic factors that produce maladaptive eating disorders and vulnerability to obesity before the clinical onset of the disease. Some of these factors have been already evidenced, including efficiency of peripheral endocrinological and metabolic processes (8, 9, 26, 27), and responsivness of the dopaminergic system to food cues (28). Here we show that interindividual variability in the feeding response to short-term HFD is also linked to the intrahypothalamic PSA-NCAM signaling. This is in line with previous works from our group showing that experimental removal of hypothalamic PSA-NCAM increases the feeding response to HFD and the body weight gain (16, 24).

After the DFTT, intrahypothalamic PSA-NCAM content was elevated in HFD-tolerant mice, while it remained low and similar to the constitutive level in HFD-intolerant mice. This result suggests that inter-individual variability exists in the ability to upregulate PSA-NCAM production in the hypothalamus in response to the metabolic challenge, and that PSA-NCAM production was stimulated in HFD-tolerant mice. The source of variation of PSA-NCAM expression between HFD-tolerant and -intolerant animals is not known. Similar intrinsic differences in healthy animals have been already reported, linking the level of prefrontal PSA-NCAM to the susceptibility for addiction-related behaviors (29). Interindividual differences in learning-induced PSA-NCAM levels in the hippocampus is also linked to learning ability in rats (30). Interestingly, acute stress that predisposes young rats to mood and anxiety disorders during adulthood, as well as chronic stress in adult, cause dramatic alteration in PSA-NCAM expression in the limbic system (31, 32). These results show that brain PSA-NCAM synthesis is affected not only by genetic heritage but also by experience. Since litter size and maternal care are early determinants of vulnerability to obesity (33–36), PSA-NCAM variation in our model might by linked to perinatal experiences. At the molecular level, biosynthesis of PSA-NCAM in adult brain requires successive biochemical reactions, which are catalyzed by enzymes including UDP-GlcNAc 2-epimerase/ManNAc kinase and polysialyltransferase 1, encoded by *Gne* and *St8sia4* genes, respectively. One can propose that these genes might be differentially upregulated on HFD depending on individuals. Gene polymorphism association studies and epigenetic research on these specific genes in murine models and in humans are thus promising research to increase our knowledge on obesity susceptibility.

In several models of neuropsychological disorders, the loss of neural PSA-NCAM alters physiological defenses, exacerbates symptoms or worsens the disease (29, 37–40), pointing out the potential protective role of PSA-NCAM against brain dysfunctions involving a plasticity-related component. In previous works, we found that removal of hypothalamic PSA is sufficient to alter short-term homeostatic responses to dietary fat and increase body weight gain on a long term (16, 24, 25), confirming that hypothalamic PSA-NCAM is critical to maintain energy homeostasis upon metabolic challenge. Together, these data suggest a causal link between hypothalamic PSA-NCAM and the propensity for obesity. Given the role of PSA-NCAM in brain plasticity (23, 41), our findings further strengthen the concept that factors controlling neural plasticity are critical in the individual susceptibility to develop overweight with obesogenic foods (22, 42, 43). Consistently, a growing body of evidence indicates that weight gain in human is associated to genes encoding plasticity-related molecules (12, 19, 20, 44–47).

Future research is now needed to understand molecular basis of inter-individual heterogeneity in hypothalamic PSA-NCAM level and to identify life stressors that influence the expression of this factor in the hypothalamus.

## Author Contributions

All authors listed have made a substantial, direct and intellectual contribution to the work, and approved it for publication.

### Conflict of Interest Statement

The authors declare that the research was conducted in the absence of any commercial or financial relationships that could be construed as a potential conflict of interest.

## Supplementary Material

Figure S1 Acute feeding response of HFD-fed mice. **(A)** Average homeostatic feeding response to HFD in 2-month old male mice (C57Bl/6JOla) (*n* = 31; ^*^*p* < 0.05, ^**^*p* < 0.01, and ^***^*p* < 0.001; one-way ANOVA for repeated measures followed by multiple comparison Dunnett *post-hoc* test vs. day 0). **(B)** Graphical representation of the individual feeding responses to 1-week HFD in 2-month old male mice (C57Bl/6JOla) (*n* = 31). **(C)** Plotting of the cumulative energy intake of mice on HFD for 1 week. Median split separated HFD-tolerant mice with low feeding response (gray; *n* = 16) and HFD-intolerant mice with high feeding response (red; *n* = 15).

## References

[1] Hill JO Peters JC. Environmental contributions to the obesity epidemic. Science (1998) 280:1371–4. 10.1126/science.280.5368.1371 9603719

[2] El-Sayed Moustafa JS Froguel P. From obesity genetics to the future of personalized obesity therapy. Nat Rev Endocrinol. (2013) 9:402–13. 10.1038/nrendo.2013.57 23529041

[3] Friedman JM A war on obesity, not the obese. Science (2003) 299:856–8. 10.1126/science.1079856 12574619

[4] Levin BE Sullivan AC. Glucose-induced norepinephrine levels and obesity resistance. Am J Physiol. (1987) 253:R475–81. 10.1152/ajpregu.1987.253.3.R475 3307459

[5] Chang S Graham B Yakubu F Lin D Peters JC Hill JO. Metabolic differences between obesity-prone and obesity-resistant rats. Am J Physiol. (1990) 259:R1103–10. 10.1152/ajpregu.1990.259.6.R1103 2260721

[6] Buchanan TA Fisler JS Underberger S Sipos GF Bray GA Whole body insulin sensitivity in Osborne-Mendel and S 5B/Pl rats eating a low- or high-fat diet. Am J Physiol. (1992) 263:R785–9. 10.1152/ajpregu.1992.263.4.R785 1415789

[7] Ji H Friedman MI. Reduced hepatocyte fatty acid oxidation in outbred rats prescreened for susceptibility to diet-induced obesity. Int J Obes. (2008) 32:1331–4. 10.1038/ijo.2008.71 18504445

[8] Pagliassotti MJ Knobel SM Shahrokhi KA Manzo AM Hill JO. Time course of adaptation to a high-fat diet in obesity-resistant and obesity-prone rats. Am J Physiol. (1994) 267:R659–64. 10.1152/ajpregu.1994.267.3.R659 8092309

[9] Dourmashkin JT Chang GQ Hill JO Gayles EC Fried SK Leibowitz SF. Model for predicting and phenotyping at normal weight the long-term propensity for obesity in Sprague-Dawley rats. Physiol Behav. (2006) 87:666–78. 10.1016/j.physbeh.2006.01.008 16513148

[10] Darling JN Ross AP Bartness TJ Parent MB. Predicting the effects of a high-energy diet on fatty liver and hippocampal-dependent memory in male rats. Obesity (2013) 21:910–7. 10.1002/oby.20167 23784893 PMC3695417

[11] Vaanholt LM Sinclair RE Mitchell SE Speakman JR. Factors influencing individual variability in high fat diet-induced weight gain in out-bred MF1 mice. Physiol Behav. (2015) 144:146–55. 10.1016/j.physbeh.2015.03.029 25817538

[12] Locke AE Kahali B Berndt SI Justice AE Pers TH Day FR. Genetic studies of body mass index yield new insights for obesity biology. Nature (2015) 518:197–206. 10.1038/nature14177 25673413 PMC4382211

[13] Hoffmann TJ Choquet H Yin J Banda Y Kvale MN Glymour M A large multi-ethnic genome-wide association study of adult body mass index identifies novel loci. Genetics (2018) 210:499–515. 10.1534/genetics.118.301479 30108127 PMC6216593

[14] Butler AA Marks DL Fan W Kuhn CM Bartolome M Cone RD. Melanocortin-4 receptor is required for acute homeostatic responses to increased dietary fat. Nat Neurosci. (2001) 4:605–11. 10.1038/88423 11369941

[15] Ellacott KL Morton GJ Woods SC Tso P Schwartz MW. Assessment of feeding behavior in laboratory mice. Cell Metab. (2010) 12:10–7. 10.1016/j.cmet.2010.06.001 20620991 PMC2916675

[16] Benani A Hryhorczuk C Gouaze A Fioramonti X Brenachot X Guissard C. Food intake adaptation to dietary fat involves PSA-dependent rewiring of the arcuate melanocortin system in mice. J Neurosci. (2012) 32:11970–9. 10.1523/JNEUROSCI.0624-12.2012 22933782 PMC6621529

[17] Schwartz MW Porte D Jr. Diabetes, obesity, and the brain. Science (2005) 307:375–9. 10.1126/science.1104344 15662002

[18] Eckel RH Kahn SE Ferrannini E Goldfine AB Nathan DM Schwartz MW. Obesity and type 2 diabetes: what can be unified and what needs to be individualized? J Clin Endocrinol Metab. (2011) 96:1654–63. 10.1210/jc.2011-0585 21602457 PMC3206399

[19] Thorleifsson G Walters GB Gudbjartsson DF Steinthorsdottir V Sulem P Helgadottir A. Genome-wide association yields new sequence variants at seven loci that associate with measures of obesity. Nat Genet. (2009) 41:18–24. 10.1038/ng.274 19079260

[20] Willer CJ Speliotes EK Loos RJ Li S Lindgren CM Heid IM. Six new loci associated with body mass index highlight a neuronal influence on body weight regulation. Nat Genet. (2009) 41:25–34. 10.1038/ng.287 19079261 PMC2695662

[21] Bouret SG Gorski JN Patterson CM Chen S Levin BE Simerly RB. Hypothalamic neural projections are permanently disrupted in diet-induced obese rats. Cell Metab. (2008) 7:179–85. 10.1016/j.cmet.2007.12.001 18249177 PMC2442478

[22] Horvath TL Sarman B Garcia-Caceres C Enriori PJ Sotonyi P Shanabrough M. Synaptic input organization of the melanocortin system predicts diet-induced hypothalamic reactive gliosis and obesity. Proc Natl Acad Sci USA. (2010) 107:14875–80. 10.1073/pnas.1004282107 20679202 PMC2930476

[23] Rutishauser U. Polysialic acid in the plasticity of the developing and adult vertebrate nervous system. Nature Rev Neurosci. (2008) 9:26–35. 10.1038/nrn2285 18059411

[24] Brenachot X Rigault C Nedelec E Laderriere A Khanam T Gouaze A. The histone acetyltransferase MOF activates hypothalamic polysialylation to prevent diet-induced obesity in mice. Mol Metab. (2014) 3:619–29. 10.1016/j.molmet.2014.05.006 25161885 PMC4142401

[25] Brenachot X Gautier T Nedelec E Deckert V Laderriere A Nuzzaci D. Brain control of plasma cholesterol involves polysialic acid molecules in the hypothalamus. Front Neurosci (2017) 11:245. 10.3389/fnins.2017.00245 28515677 PMC5414510

[26] Levin BE Dunn-Meynell AA Balkan B Keesey RE. Selective breeding for diet-induced obesity and resistance in Sprague-Dawley rats. Am J Physiol. (1997) 273:R725–30. 10.1152/ajpregu.1997.273.2.R725 9277561

[27] Perez-Echarri N Perez-Matute P Martinez JA Marti A Moreno-Aliaga MJ Serum and gene expression levels of leptin and adiponectin in rats susceptible or resistant to diet-induced obesity. J Physiol Biochem. (2005) 61:333–42. 10.1007/BF03167050 16180331

[28] Rada P Bocarsly ME Barson JR Hoebel BG Leibowitz SF. Reduced accumbens dopamine in Sprague-Dawley rats prone to overeating a fat-rich diet. Physiol Behav. (2010) 101:394–400. 10.1016/j.physbeh.2010.07.005 20643155 PMC2930885

[29] Barker JM Torregrossa MM Taylor JR. Low prefrontal PSA-NCAM confers risk for alcoholism-related behavior. Nat Neurosci. (2012) 15:1356–8. 10.1038/nn.3194 22922785 PMC3629946

[30] Sandi C Cordero MI Merino JJ Kruyt ND Regan CM Murphy KJ. Neurobiological and endocrine correlates of individual differences in spatial learning ability. Learn Mem. (2004) 11:244–52. 10.1101/lm.73904 15169853 PMC419726

[31] Sandi C Merino JJ Cordero MI Touyarot K Venero C. Effects of chronic stress on contextual fear conditioning and the hippocampal expression of the neural cell adhesion molecule, its polysialylation, and L1. Neuroscience (2001) 102:329–39. 10.1016/S0306-4522(00)00484-X 11166119

[32] Tsoory M Guterman A Richter-Levin G. Exposure to stressors during juvenility disrupts development-related alterations in the PSA-NCAM to NCAM expression ratio: potential relevance for mood and anxiety disorders. Neuropsychopharmacology (2008) 33:378–93. 10.1038/sj.npp.1301397 17429411

[33] Roberts JL Whittington FM Enser M. Effects of litter size and subsequent gold-thioglucose-induced obesity on adipose tissue weight, distribution and cellularity in male and female mice: an age study. Br J Nutr. (1988) 59:519–33. 10.1079/BJN19880061 3134938

[34] Voits M Forster S Rodel S Voigt JP Plagemann A Fink H. Obesity induced by unspecific early postnatal overfeeding in male and female rats: hypophagic effect of CCK-8S. Naunyn Schmiedebergs Arch Pharmacol. (1996) 354:374–8. 10.1007/BF00171071 8878070

[35] Mcewen BS. Understanding the potency of stressful early life experiences on brain and body function. Metabolism (2008) 57 (Suppl. 2):S11–5. 10.1016/j.metabol.2008.07.006 18803958 PMC2567059

[36] Clarke MA Stefanidis A Spencer SJ. Postnatal overfeeding leads to obesity and exacerbated febrile responses to lipopolysaccharide throughout life. J Neuroendocrinol. (2012) 24:511–24. 10.1111/j.1365-2826.2011.02269.x 22175701

[37] El Maarouf A Kolesnikov Y Pasternak G Rutishauser U. Polysialic acid-induced plasticity reduces neuropathic insult to the central nervous system. Proc Natl Acad Sci USA. (2005) 102:11516–20. 10.1073/pnas.0504718102 16055555 PMC1183577

[38] Lopez-Fernandez MA Montaron MF Varea E Rougon G Venero C Abrous DN. Upregulation of polysialylated neural cell adhesion molecule in the dorsal hippocampus after contextual fear conditioning is involved in long-term memory formation. J Neurosci. (2007) 27:4552–61. 10.1523/JNEUROSCI.0396-07.2007 17460068 PMC6673006

[39] Markram K Lopez Fernandez MA Abrous DN Sandi C Amygdala upregulation of NCAM polysialylation induced by auditory fear conditioning is not required for memory formation, but plays a role in fear extinction. Neurobiol Learn Mem. (2007) 87:573–82. 10.1016/j.nlm.2006.11.007 17223582

[40] Mccall T Weil ZM Nacher J Bloss EB El Maarouf A Rutishauser U. Depletion of polysialic acid from neural cell adhesion molecule (PSA-NCAM) increases CA3 dendritic arborization and increases vulnerability to excitotoxicity. Exp Neurol. (2013) 241:5–12. 10.1016/j.expneurol.2012.11.028 23219884 PMC3570583

[41] Durbec P Cremer H. Revisiting the function of PSA-NCAM in the nervous system. Mol Neurobiol. (2001) 24:53–64. 10.1385/MN:24:1-3:053 11831554

[42] Dietrich MO Horvath TL. Hypothalamic control of energy balance: insights into the role of synaptic plasticity. Trends Neurosci. (2013) 36:65–73. 10.1016/j.tins.2012.12.005 23318157

[43] Nuzzaci D Laderriere A Lemoine A Nedelec E Penicaud L Rigault C. Plasticity of the melanocortin system: determinants and possible consequences on food intake. Front Endocrinol (Lausanne). (2015) 6:143. 10.3389/fendo.2015.00143 26441833 PMC4568417

[44] Speliotes EK Willer CJ Berndt SI Monda KL Thorleifsson G Jackson AU. Association analyses of 249,796 individuals reveal 18 new loci associated with body mass index. Nat Genet. (2010) 42:937–48. 10.1038/ng.686 20935630 PMC3014648

[45] Rathjen T Yan X Kononenko NL Ku MC Song K Ferrarese L. Regulation of body weight and energy homeostasis by neuronal cell adhesion molecule 1. Nat Neurosci. (2017) 20:1096–103. 10.1038/nn.4590 28628102 PMC5533218

[46] Bochukova EG Lawler K Croizier S Keogh JM Patel N Strohbehn G. A transcriptomic signature of the hypothalamic response to fasting and bdnf deficiency in prader-willi syndrome. Cell Rep. (2018) 22:3401–8. 10.1016/j.celrep.2018.03.018 29590610 PMC5896230

[47] Turcot V Lu Y Highland HM Schurmann C Justice AE Fine RS Protein-altering variants associated with body mass index implicate pathways that control energy intake and expenditure in obesity. Nat Genet. (2018) 50:26–41. 10.1038/s41588-017-0011-x 29273807 PMC5945951

