# Supplementary figures and images for: Lack of Hypothalamus Polysialylation Inducibility Correlates With Maladaptive Eating Behaviors and Predisposition to Obesity
Source: Front Nutr. 2018 Dec 10;5:125. doi: 10.3389/fnut.2018.00125 (PMC6295648; doi:10.3389/fnut.2018.00125)

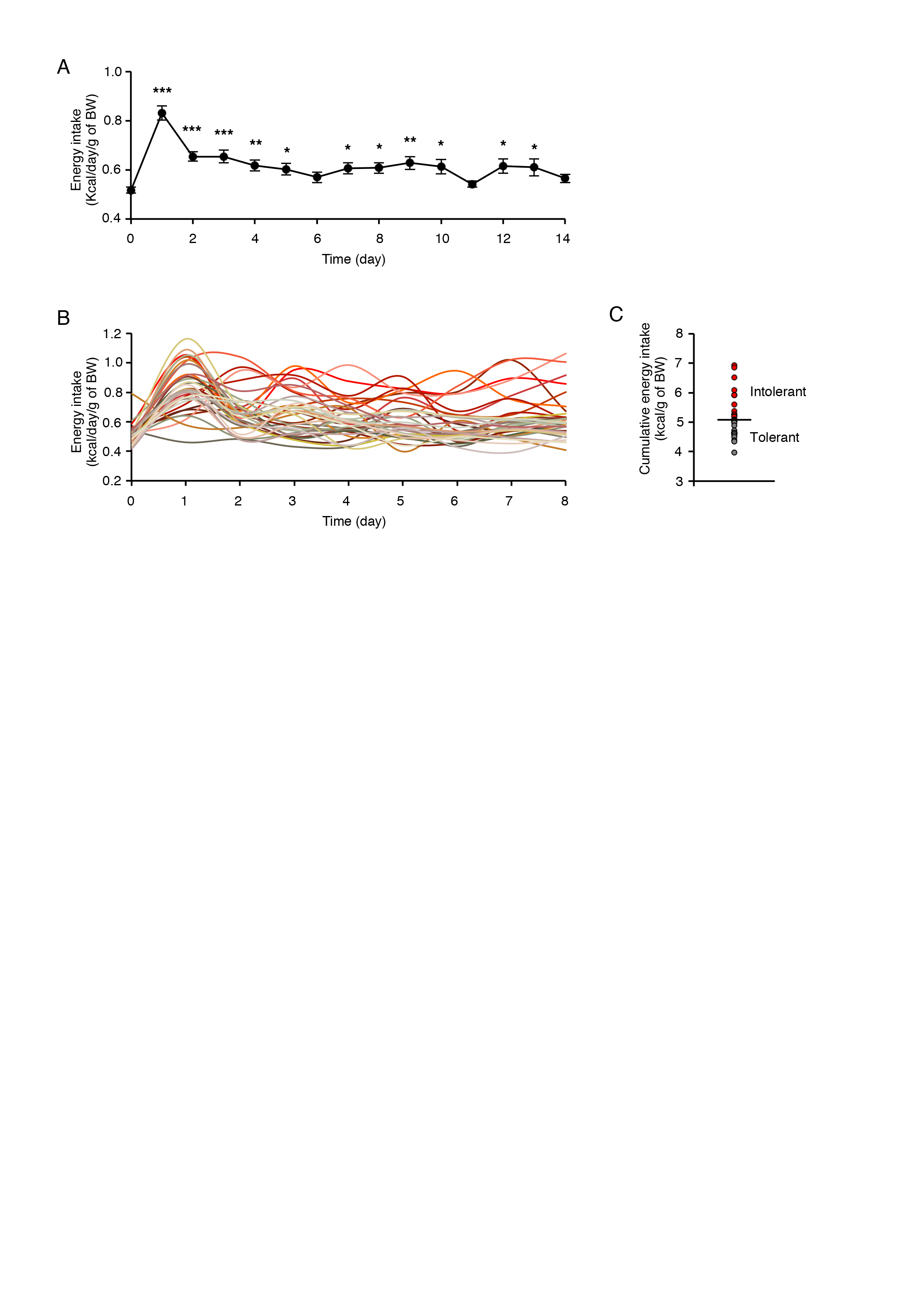

Supplement: Figure S1 — Acute feeding response of HFD-fed mice. (A) Average homeostatic feeding response to HFD in 2-month old male mice (C57Bl/6JOla) (n = 31; *p < 0.05, **p < 0.01, and ***p < 0.001; one-way ANOVA for repeated measures followed by multiple comparison Dunnett post-hoc test vs. day 0). (B) Graphical representation of the individual feeding responses to 1-week HFD in 2-month old male mice (C57Bl/6JOla) (n = 31). (C) Plotting of the cumulative energy intake of mice on HFD for 1 week. Median split separated HFD-tolerant mice with low feeding response (gray; n = 16) and HFD-intolerant mice with high feeding response (red; n = 15). [file Image_1.JPEG]
